# The Potential of Trigona Honey as a Supplementary Therapy for Vascular Cognitive Impairment in Patients with Acute Ischaemic Stroke

**DOI:** 10.21315/mjms-05-2025-359

**Published:** 2025-10-31

**Authors:** Mohd Haniff Abu Zarim, Anis Kausar Ghazali, Sanihah Abdul Halim, Engku Nur Syafirah Engku Abd Rahman, Manal Abdel Haleem A. Abusalah, Yean Yean Chan, Sabarisah Hashim

**Affiliations:** 1 Department of Internal Medicine, School of Medical Sciences, Health Campus, Universiti Sains Malaysia, Kelantan, Malaysia; 2 Hospital Universiti Sains Malaysia, Health Campus, Universiti Sains Malaysia, Kelantan, Malaysia; 3 Unit of Biostatistics and Research Methodology, School of Medical Sciences, Health Campus, Universiti Sains Malaysia, Kelantan, Malaysia; 4 Department of Medical Microbiology and Parasitology, School of Medical Sciences, Health Campus, Universiti Sains Malaysia, Kelantan, Malaysia; 5 Department of Neurosciences School of Medical Sciences, Health Campus, Universiti Sains Malaysia, Kelantan, Malaysia

**Keywords:** vascular cognitive impairment, ischaemic stroke, MOCA, MRS, Trigona honey

## Abstract

**Background:**

Vascular cognitive impairment (VCI) is a common, yet often underrecognised, consequence of acute ischaemic stroke. Despite its prevalence, therapeutic strategies targeting VCI remain limited. Trigona honey, known for its antioxidant and neuroprotective properties, may offer a novel adjunctive intervention to improve cognitive outcomes after stroke.

**Methods:**

This randomised controlled study involved 48 patients diagnosed with acute ischaemic stroke. Participants were randomly assigned to two equal groups: an intervention group receiving daily supplementation with Trigona honey (*n* = 24) and a control group without supplementation (*n* = 24). Cognitive function was evaluated using the Montreal Cognitive Assessment (MOCA), and global disability was measured using the Modified Rankin Scale (MRS). Assessments were conducted at baseline and after three months of intervention.

**Results:**

At baseline, 66.7% of participants were male, with most exhibiting mild cognitive impairment. After three months, the intervention group showed significant improvement in MOCA scores (from 20.08 ± 4.53 to 24.33 ± 3.64), while the control group showed only a modest increase (from 18.42 ± 5.12 to 19.67 ± 6.31). The between-group difference in MOCA scores was 4.25 (95% CI: 3.07, 5.43; *P* < 0.001). Functional status also improved significantly in the Trigona honey group, with a mean change in MRS score of −1.29 (95% CI: −1.71, −0.87; *P* < 0.001), indicating enhanced independence compared to the control group.

**Conclusion:**

Trigona honey supplementation significantly improved cognitive performance and functional outcomes in patients with VCI following ischaemic stroke, supporting its role as a promising adjunct in stroke rehabilitation.

## Introduction

Vascular cognitive impairment (VCI) is a prevalent and potentially disabling consequence of stroke, affecting a significant proportion of stroke survivors (1). It is estimated that nearly one-third of individuals worldwide will experience a stroke, dementia, or both, with up to 64% of stroke survivors developing some degree of cognitive impairment and approximately one-third progressing to dementia (2, 3). Cognitive deficits in stroke survivors can affect various domains, including memory, attention, executive function, language, and visuospatial abilities, significantly impacting daily living and functional independence (4). Despite its clinical significance, post-stroke cognitive impairment remains underdiagnosed, and effective therapeutic interventions are limited. Stroke survivors and their caregivers frequently identify cognitive deficits as a primary concern due to their association with increased morbidity, prolonged hospitalisation, higher dependency, and diminished quality of life (5, 6).

VCI is characterised by cognitive dysfunction resulting from cerebrovascular pathology, encompassing both large-vessel occlusions and small-vessel disease, as well as mixed neurodegenerative conditions, such as Alzheimer’s disease (4, 5, 7). Established risk factors for VCI include ageing, hypertension, diabetes, hyperlipidaemia, hyperuricaemia, carotid atherosclerosis, and smoking (4). Given that many of these vascular risk factors are modifiable, early intervention may help prevent, delay, or mitigate the progression of cognitive decline (7). However, despite advances in stroke management, effective treatments targeting post-stroke cognitive impairment remain elusive (4).

Current pharmacological options, such as acetylcholinesterase inhibitors (donepezil, rivastigmine, galantamine) and N-methyl-D-aspartate (NMDA) receptor antagonists (memantine), have demonstrated modest cognitive benefits but lack consistent improvements in functional outcomes (8). While non-pharmacological strategies, such as cognitive training, lifestyle modifications, and vascular risk factor control, play a role in managing VCI, large-scale clinical trials are still lacking (9).

Recently, there has been growing interest in the potential role of natural bioactive compounds to promote cognitive recovery. Of these, honey, a natural product derived from honeybees (*Apis* spp.) and stingless bees (*Meliponini* spp.) that is known for its antioxidant, anti-inflammatory, and neuroprotective properties, has emerged as a promising candidate (10–14). In particular, Trigona honey, also known as Kelulut honey, produced by the stingless bee *Heterotrigona itama*, has garnered attention due to its superior antioxidant activity compared to conventional honey varieties, such as Manuka honey (15). This honey is rich in bioactive compounds, including phenolic acids, flavonoids, organic acids, amino acids, and essential minerals, which have been associated with various health benefits, including neuroprotection (13, 16).

Studies suggest that certain components of Trigona honey, such as phenylalanine, may upregulate brain-derived neurotrophic factor (BDNF) and inositol 1,4,5-triphosphate receptor type 1 (ITPR1), both of which are implicated in synaptic plasticity and cognitive enhancement (11). Furthermore, experimental research has shown that honey supplementation improves spatial memory, learning, and neuronal survival while also exhibiting anxiolytic and antidepressant-like effects in animal models of neurodegeneration (13, 15, 17).

Despite promising preclinical evidence, the therapeutic potential of Trigona honey in stroke patients remains largely unexplored. While honey has traditionally been used for medicinal purposes, there is a critical gap in research assessing its role in preventing post-stroke cognitive decline and improving functional recovery. Given its potent antioxidant and neuroprotective effects, Trigona honey could serve as a promising adjunctive therapy to support cognitive rehabilitation in stroke survivors (18, 19). This study aims to investigate the effects of Trigona honey supplementation on cognitive function and functional recovery among patients with acute ischaemic stroke, providing new insights into its potential role in mitigating VCI.

## Methods

### Study Design and Participant Recruitment

**Figure 1:**
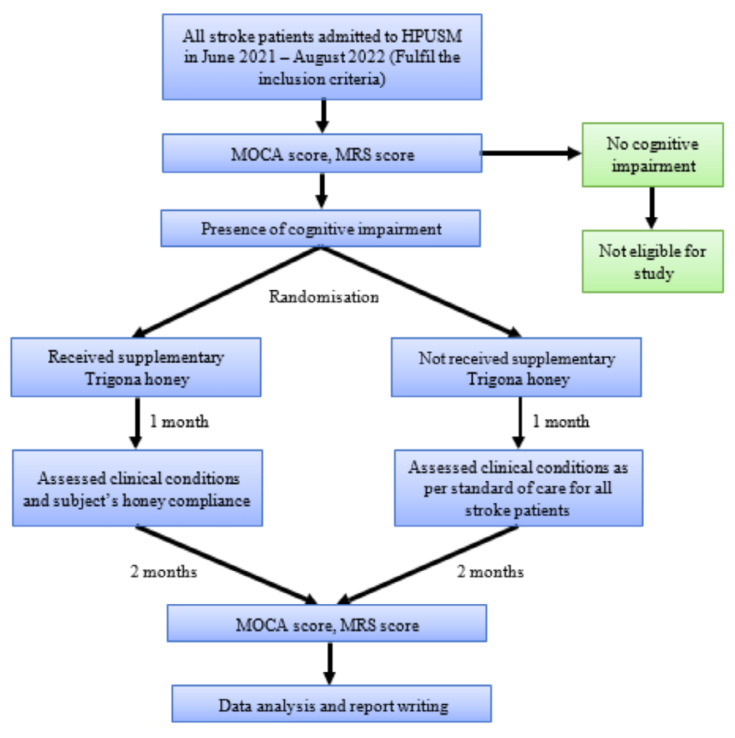
Recruitment of participants for intervention (Trigona honey) and control groups

This study was a randomised controlled trial conducted to evaluate the effects of Trigona honey supplementation in mitigating VCI progression in acute ischaemic stroke patients (Figure 1). Participants aged 20 to 70 years who were hospitalised within 72 hours post-stroke were recruited between June 2021 and August 2022 at Universiti Sains Malaysia Specialist Hospital, Kelantan, Malaysia. Eligibility criteria included a Montreal Cognitive Assessment (MOCA) score below 26. Patients with a history of progressive cognitive impairment, severe aphasia, uncontrolled diabetes mellitus (capillary blood glucose > 10 mmol/L post-stroke onset), severe stroke, or comatose state were excluded. Informed consent was obtained from all participants or their legal representatives.

### Randomisation and Intervention

Eligible participants were randomised into two groups: the intervention group (*n* = 24) received Trigona honey supplementation (10 g twice daily) for 12 weeks, while the control group (*n* = 24) did not receive supplementation. The selected dosage of 20 g/day was based on traditional consumption practices.

### Outcome Measures

Cognitive impairment was assessed using the MOCA test at baseline and after three months. The MOCA, a 30-point cognitive screening tool, evaluates visuospatial/executive function, naming, attention, language, abstraction, memory, and orientation. Patients were classified based on MOCA scores: mild cognitive impairment (> 25), moderate VCI (18 to 25), and severe VCI (0 to 9). Disability and functional independence were assessed using the Modified Rankin Scale (MRS), a structured scale ranging from 0 (no symptoms) to 6 (death). Additional assessments included the National Institutes of Health Stroke Scale (NIHSS), a 15-item neurological statistical analysis evaluation tool measuring deficits such as consciousness, language, neglect, motor strength, and sensory loss. NIHSS scores range from 0 (no deficits) to 42 (severe deficits). The Trial of Org 10172 in Acute Stroke Treatment (TOAST) criteria, is a prevalent system for categorising ischaemic stroke subtypes by aetiology into five main types: large-artery atherosclerosis, cardioembolism, small-vessel occlusion, determined aetiology stroke, and undetermined aetiology stroke.

### Statistical Analysis

All statistical analyses were performed using SPSS version 27. Descriptive statistics summarised sociodemographic and clinical characteristics. Categorical variables were presented as frequencies (*n*) and percentages (%), while numerical variables were reported as means with standard deviations (SD) or medians with interquartile ranges (IQR), depending on data normality. Repeated measures analysis of variance (ANOVA) was employed to compare MOCA scores between the intervention and control groups at baseline and 6 months post intervention. Similarly, ANOVA was used to evaluate changes in MRS scores within the intervention group.

## Results

### Sociodemographic Characteristics of The Subjects

A total of 48 subjects (24 in the intervention group and 24 in the control group) aged between 35 and 70 years participated in this study, with a mean age of 58.85 ± 9.21 years. The majority were Malay (97.9%) and male (66.7%). Common comorbidities included hyperlipidaemia (100%), hypertension (95.8%), diabetes mellitus (62.5%), and ischaemic heart disease (35.4%) (Table 1). The dataset used and analysed in this study is available from the corresponding author upon reasonable request. However, due to licensing restrictions, it is not publicly accessible.

### Clinical Characteristics of The Subjects

Subjects were classified according to the TOAST criteria for ischaemic stroke aetiology. The majority had small-vessel occlusions (64.6%), followed by large-artery atherosclerosis (18.8%) and cardioembolic stroke (12.5%). Most subjects presented with mild neurological deficits (NIHSS score 1 to 4, 75%), while 22.9% had moderate deficits (NIHSS score 5 to 14), and 2.1% had severe deficits (NIHSS score 15 to 24) (Table 2). Regarding medication use, none of the subjects underwent thrombolysis. The majority were on antiplatelet therapy (93.8%), anticoagulants (10.4%), antidiabetic medications (68.1%), antihypertensive medications (100%), and statins (100%).

### Cognitive Impairment and Functional Independence at Baseline and After Three Months

Cognitive impairment was assessed using the MOCA scale and categorised into normal (> 25), mild (18 to 25), moderate (10 to 17), and severe (< 10). In the Trigona honey group, most subjects had mild cognitive impairment at baseline (79.2%), followed by moderate impairment (20.8%). After three months, cognitive function improved, with 45.8% classified as normal, 50% as mild, and 4.2% as moderate. In contrast, in the control group, 66.7% of subjects initially had mild impairment, 25% had moderate impairment, and 8.3% had severe impairment. After three months, cognitive function improved to normal (16.7%), mild (54.2%), moderate (20.8%), and severe (8.3%) (Table 3).

Functional independence was measured using the MRS. In the Trigona honey group, baseline MRS scores indicated mild disability in 54.2%, moderate disability in 25%, and severe disability in 20.8% of patients. After three months, improvement was observed, with 62.5% of patients achieving normal functions, 20.8% having mild disability, and 16.7% having moderate disability. The control group initially had 45.8% of patients with moderate disability, 33.4% with mild disability, and 20.8% with severe disability. After three months, 45.8% had normal function, 41.7% had mild disability, 8.3% had moderate disability, and 4.2% had severe disability.

### Comparison of Cognitive Impairment (MOCA Score) Between Groups

In the Trigona honey group, the mean MOCA score increased from 20.08 ± 4.53 at baseline to 24.33 ± 3.64 after three months, with a significant mean difference of 4.25 (95% CI: 3.07, 5.43; *P* < 0.001). Meanwhile, the control group showed an increase from 18.42 ± 5.12 to 19.67 ± 6.31, with a nonsignificant mean difference of 1.25 (95% CI: −0.63, 3.13; *P* = 0.182). A repeated measures ANOVA confirmed a significant difference between the two groups (*P* = 0.022) (Table 4). These findings suggest that Trigona honey supplementation significantly improves cognitive function in patients with acute ischaemic stroke.

### VCI Resolution After Three Months

MOCA scores were further categorised into the presence of VCI (MOCA ≤ 25) and resolution of VCI (MOCA > 25). In the Trigona honey group, all subjects had VCI at baseline (100%), but after three months, 11 subjects (45.8%) had achieved VCI resolution (*P* = 0.001). Conversely, in the control group, all subjects had VCI at baseline (100%), and after three months, only four subjects (16.7%) showed VCI resolution (*P* = 0.125). The overall comparison showed a significant improvement in VCI resolution in the Trigona honey group (*P* < 0.001) (Table 5).

### Comparison of Functional Independence (MRS Score) in The Trigona Honey Group

In the Trigona honey group, the mean MRS score significantly improved from 1.92 ± 1.21 at baseline to 0.63 ± 0.97 after three months, with a mean difference of −1.29 (95% CI: −1.71, −0.87; *P* < 0.001) (Table 6). This suggests that Trigona honey supplementation contributes to significant improvements in global disability and functional independence among acute ischaemic stroke patients with VCI. These findings highlight the potential therapeutic benefits of Trigona honey in improving cognitive function, reducing VCI, and enhancing functional recovery in stroke patients.

## Discussion

In our study, 48 subjects (24 in the intervention group and 24 in the control group) aged 35 to 70 years (mean age: 58.85 ± 9.21 years) participated. The majority were Malay (97.9%) and male (66.7%), with prevalent comorbidities including hyperlipidaemia (100.0%), hypertension (95.8%), diabetes mellitus (62.5%), and ischaemic heart disease (35.4%) (Table 1). No significant baseline differences in sociodemographic characteristics were observed between the intervention and control groups. These findings align with Malaysian national data, particularly the Acute Stroke Registry Malaysia (2010 to 2014), which reported a mean stroke patient age of 62.7 ± 12.5 years and a predominance of male patients (55.0%) (20). The ethnic distribution in this study corresponds to Kelantan’s demographic composition, where Malays constitute 95.7% of the population (21). Similar trends have been reported in studies conducted in the United States, the United Kingdom, and China (22–25).

Vascular risk factors, including hyperlipidaemia (100.0%), hypertension (95.8%), diabetes mellitus (62.5%), and smoking (50.0%), are known contributors to VCI. The interplay between cholesterol levels, small-vessel disease, stroke, cognitive impairment, and dementia remains complex and not fully understood. In the absence of definitive guidelines, managing vascular risk factors according to established cardiovascular prevention strategies remains prudent (22). Hypertension, a predominant risk factor, has been associated with an 8.4% higher prevalence among females in Malaysia’s National Stroke Registry (20). Blood pressure management remains crucial, with recommended targets of ≤ 140/90 mmHg and additional benefits observed at ≤ 130 mmHg. Lifestyle modifications, including salt reduction, weight loss, and regular physical activity, are essential adjuncts. The role of antiplatelet agents in primary prevention remains controversial, with no evidence supporting stroke risk reduction in low-risk populations (23).

Stroke subtypes were classified using TOAST criteria, revealing a predominance of small-vessel occlusion (64.6%), followed by large-artery atherosclerosis (18.8%) and cardio embolism (12.5%). The severity of neurological deficits, assessed using the NIHSS, indicated that most subjects had mild deficits (NIHSS 1 to 4: 75%), followed by moderate deficits (NIHSS 5 to 14: 22.9%) and severe deficits (NIHSS 15 to 24: 2.1%) (Table 2). At baseline, all subjects were on antihypertensive therapy (100.0%), antiplatelets (93.8%), statins (100.0%), and antidiabetic medications (68.1%), with no significant clinical differences between the intervention and control groups.

The study’s primary objective was to evaluate the effect of Trigona honey supplementation on cognitive function using the MOCA score. Baseline cognitive impairment levels in the Trigona honey group were mild (79.2%) and moderate (20.8%). After three months, cognitive improvement was observed, with 45.8% achieving normal cognition, 50.0% retaining mild impairment, and 4.2% remaining in the moderate category. In contrast, the control group exhibited minimal improvement, with 16.7% achieving normal cognition, while 54.2%, 20.8%, and 8.3% remained in the mild, moderate, and severe impairment categories, respectively.

The mean MOCA score in the Trigona honey group improved from 20.08 ± 4.53 to 24.33 ± 3.64, yielding a significant mean difference of 4.25 (95% CI: 3.07, 5.43; *P* < 0.001). Meanwhile, the control group exhibited a nonsignificant increase from 18.42 ± 5.12 to 19.67 ± 6.31 (mean difference: 1.25; 95% CI: −0.63, 3.13; *P* = 0.182). Repeated measures of ANOVA confirmed a statistically significant cognitive impairment in the intervention group (*P* = 0.022) (Table 4). Furthermore, VCI resolution (MOCA > 25) was achieved in 45.8% of Trigona honey recipients (*P* = 0.001), compared to only 16.7% in the control group (*P* = 0.125), demonstrating a significant intergroup difference (*P* < 0.001) (Table 5).

These findings are consistent with previous research on honey’s cognitive benefits. A Malaysian study by Yahaya et al. (26) demonstrated improved verbal memory in schizophrenia patients following Tualang honey supplementation (*P* < 0.05). Similarly, Othman et al. (27) reported enhanced immediate memory in postmenopausal women receiving Tualang honey compared to hormonal therapy. Al-Himyari et al. (28) conducted a randomised, placebo-controlled study on 2,893 elderly individuals and found that honey supplementation significantly reduced dementia incidence over five years (*P* < 0.05). Compared to conventional pharmacotherapy for VCI, our findings align with the efficacy of cholinesterase inhibitors. A 2021 network meta-analysis indicated that donepezil (5 mg and 10 mg) and galantamine confer modest cognitive benefits in VCI, although the effect size remains clinically marginal (29).

Our study suggests that Trigona honey may offer a comparable or superior alternative to existing pharmacological interventions, warranting further investigation through larger, long-term trials. This study has several notable strengths. The randomised controlled trial design effectively minimises potential biases and enhances the robustness of causal inferences. Additionally, the employment of validated cognitive and functional outcome measures, specifically the MOCA and the MRS, ensures greater reliability and clinical relevance of the findings. Importantly, this investigation is among the first clinical trials to evaluate the impact of Trigona honey supplementation on VCI following ischaemic stroke, thereby contributing valuable insights to stroke rehabilitation research.

Nevertheless, certain limitations warrant consideration. The relatively small sample size may constrain the generalizability of our results. The three-month follow-up period, while adequate for capturing short-term improvements, did not address the durability of cognitive and functional gains over longer durations. Furthermore, unmeasured confounders, such as variations in participants’ nutrition and lifestyle factors, may have influenced the observed outcomes.

The cognitive improvements observed in this study could be attributed in part to Trigona honey’s neuroprotective characteristics. This honey contains phenolic acids, flavonoids, and amino acids, all of which have powerful antioxidant and anti-inflammatory properties. These bioactive substances may protect neuronal integrity and function by reducing oxidative stress and inhibiting neuroinflammation. Specific components, such as phenylalanine, have been shown in studies to enhance BDNF and inositol 1,4,5-triphosphate receptor type 1 (ITPR1), both of which are required for synaptic plasticity and cognitive enhancement (30, 31). These pathways provide a credible biological basis for our participants’ improvements in a variety of cognitive domains.

Beyond cognitive improvements, honey exhibits antidepressant-like effects, a critical consideration for post-stroke patients, who frequently experience depression. Evidence suggests that honey modulates monoaminergic neurotransmission by enhancing serotonergic and dopaminergic pathways while regulating the hypothalamic-pituitary-adrenal axis, collectively alleviating depressive symptoms. These neurobiological effects are thought to derive from honey’s rich phenolic and flavonoid content, which reduces oxidative stress and neuroinflammation while augmenting BDNF expression—central to both depression recovery and cognitive resilience (19). Malaysian studies further affirm the neuroprotective and mood-stabilising potential of stingless bee honey, underscoring its promise as a complementary intervention for neurological and psychiatric conditions. Considering the well-established link between post-stroke depression and poorer cognitive and functional outcomes, the antidepressant properties of Trigona honey may represent an additional mechanism contributing to enhanced rehabilitation in VCI (10).

Future research should consider larger, multicentre randomised controlled trials with extended follow-up spans to validate these findings and assess long-term efficacy. Investigations into optimal dosing regimens, safety profiles, and potential synergistic effects with existing therapies are also necessary. Moreover, mechanistic studies at the molecular and cellular levels will be instrumental in elucidating the pathways by which Trigona honey imparts neuroprotection, thereby expanding its therapeutic potential in VCI.

## Conclusion

In conclusion, Trigona honey supplementation demonstrated significant cognitive benefits in ischaemic stroke patients with VCI, as evidenced by improvements in MOCA scores and VCI resolution rates. These findings suggest a potential role for natural interventions in cognitive rehabilitation, underscoring the need for future research into Trigona honey’s long-term efficacy and mechanisms of action.

## Figures and Tables

**Table 1:** Sociodemographic data of the subjects (*n* = 48)

Variables	*n* (%)		

	Trigona honey (*n* = 24)	Control (*n* = 24)	Total (*n* = 48)
**Gender**			
Male	17 (70.8)	15 (62.5)	32 (66.7)
Female	7 (29.2)	9 (37.5)	16 (33.3)
**Age (years)** *	57.21 (9.78)*	60.50 (8.49)*	58.85 (9.21)*
**Ethnicity**			
Malay	24 (100.0)	23 (95.8)	47 (97.9)
Chinese	0 (0.0)	1 (4.2)	1 (2.1)
**Smoking status**			
Yes	13 (54.1)	9 (37.5)	22 (45.8)
No	10 (41.7)	14 (58.3)	24 (50.0)
Ex-smoker	1 (4.2)	1 (4.2)	2 (4.2)
**Diabetes mellitus (DM)**			
Yes	14 (58.3)	16 (66.7)	30 (62.5)
No	10 (41.7)	8 (33.3)	18 (37.5)
**Hypertension (HPT)**			
Yes	22 (91.7)	24 (100.0)	46 (95.8)
No	2 (8.3)	0 (0.0)	2 (4.2)
**Hyperlipidaemia (HPL)**			
Yes	24 (100.0)	24 (100.0)	48 (100.0)
No	0 (0.0)	0 (0.0)	0 (0.0)
**Ischaemic heart disease (IHD)**			
Yes	10 (41.7)	7 (29.2)	17 (35.4)
No	14 (58.3)	17 (70.8)	31 (64.6)

* Descriptive statistics data reported in mean (SD); *n* = frequency; % = percentage; SD = standard deviation

**Table 2:** Clinical characteristics of the subjects

Variables	*n* (%)		

	Trigona honey (*n* = 24)	Control (*n* = 24)	Total (*n* = 48)
**Stroke subtypes (TOAST)**			
Large-artery atherosclerosis	3 (12.5)	6 (25.0)	9 (18.8)
Cardioembolic	2 (8.3)	4 (16.7)	6 (12.5)
Small-vessel occlusion	18 (75.0)	13 (54.2)	31 (64.6)
Stroke of other determined aetiology	0 (0.0)	1 (4.2)	1 (2.1)
Stroke of undetermined aetiology	1 (4.2)	0 (0.0)	1 (2.1)
**Clinical stroke subtypes (OCSP)**			
TACI	0 (0.0)	0 (0.0)	0 (0.0)
PACI	2 (8.3)	3 (12.5)	5 (10.4)
POCI	2 (8.3)	4 (16.7)	6 (12.5)
LACI	20 (83.4)	17 (70.8)	37 (77.1)
**NIHSS**			
Mild	17 (70.8)	19 (79.2)	36 (75.0)
Moderate	6 (25.0)	5 (20.8)	11 (22.9)
Severe	1 (4.2)	0 (0.0)	1 (2.1)
**Thrombolysis**			
Yes	0(0.0)	0 (0.0)	(0.0)
No	24 (100.0)	24 (100.0)	48 (100.0)
**Anti-platelet**			
Yes	22 (91.7)	23 (95.8)	45 (93.8)
No	2 (8.3)	1 (4.2)	3 (6.3)
**Anti-coagulant**			
Yes	3 (12.5)	2 (8.3)	5 (10.4)
No	21 (87.5)	22 (91.7)	43 (89.6)
**Anti-diabetic**			
Yes	13 (56.5)	19 (79.2)	32 (68.1)
No	10 (43.5)	5 (20.8)	15 (31.9)
**Anti-hypertension**			
Yes	24 (100.0)	24 (100.0)	48 (100.0)
No	0 (0.0)	0 (0.0)	0 (0.0)
**Statin**			
Yes	24 (100.0)	24 (100.0)	48 (100.0)
No	0 (0.0)	0 (0.0)	0 (0.0)
**Multivitamin**			
Yes	0 (0.0)	0 (0.0)	0 (0.0)
No	24 (100.0)	24 (100.0)	48 (100.0)

Descriptive statistics; TOAST=Trial of ORG 10,172 in Acute Stroke Treatment; NIHSS = National Institute of Health Stroke Scale; OCSP = Oxfordshire Community Stroke Project; TACI = total anterior circulation infarcts; PACI = partial anterior circulation infarcts; POCI = posterior circulation infarcts; LACI = lacunar infarcts

**Table 3:** Level of MOCA and MRS at baseline and three months among Trigona honey group and control group

Variables	*n* (%)			

	Trigona honey (*n* = 24)		Control (*n* = 24)	

	Baseline	3 months	Baseline	3 months
**MOCA level**				
Normal	0 (0.0)	11 (45.8)	0 (0.0)	4 (16.7)
Mild	19 (79.2)	12 (50.0)	16 (66.7)	13 (54.2)
Moderate	5 (20.8)	1 (4.2)	6 (25.0)	5 (20.8)
Severe	0 (0.0)	0 (0.0)	2 (8.3)	2 (8.3)
**MRS level**				
Normal	0 (0.0)	15 (62.5)	0 (0.0)	11 (45.8)
Mild	13 (54.2)	5 (20.8)	8 (33.4)	10 (41.7)
Moderate	6 (25.0)	4 (16.7)	11 (45.8)	2 (8.3)
Severe	5 (20.8)	0 (0.0)	5 (20.8)	1 (4.2)

* Descriptive statistics;

MOCA = Montreal Cognitive Assessment; MRS = Modified Ranking Scale;

* significance at *P* < 0.05

**Table 4:** Comparison of mean MOCA score at baseline and three months among Trigona honey group and control group

Variables	Mean (SD)		Mean difference (95% CI)	*P* (time)^a^	*P* (group)^b^

	Baseline	3 months therapy			
**MOCA score**					
Trigona honey	20.08 (4.53)	24.33 (3.64)	4.25 (3.07, 5.43)	< 0.001*	0.022*
Control	18.42 (5.12)	19.67 (6.31)	1.25 (−0.63, 3.13)	0.182	

a *P*-value assessed by paired *t*-test;

b Repeated measure ANOVA test;

CI = confidence interval; MOCA = Montreal Cognitive Assessment;

* significant at *P* < 0.05

**Table 5:** Comparison of vascular cognitive impairment (based on MOCA score ≤ 25) at baseline and three months among Trigona honey group and control group

Variables	Trigona honey		*P*-value^a^	Control		*P*-value^a^
	
	Baseline	3 months		Baseline	3 months	
**MOCA**						
VCI (≤ 25)	24 (100.0)	13 (54.2)	0.001^*^	24 (100.0)	20 (83.3)	0.125
No VCI (> 25)	0 (0.0)	11 (45.8)		0 (0.0)	4 (16.7)	

Data in frequency (*n*) and percentage (%); VCI: MOCA ≤ 25, No VCI MOCA > 25, abnormal; MOCA = Montreal Cognitive Assessment; VCI = Vascular Cognitive Impairment;

a McNemar test

**Table 6:** Comparison of mean MRS score at baseline and three months among Trigona honey group and control group

Variables	Mean (SD)		Mean difference (95% CI)	*P* (time)^a^	*P* (group)^b^

	Baseline	3 months therapy			
**MRS Score**					
Trigona honey	1.92 (1.21)	0.63 (0.97)	−1.29 (−1.71, −0.87)	< 0.001*	0.439
Control	2.21 (1.14)	0.75 (0.94)	−1.46 (−1.97, −0.97)	< 0.001*	

a *P*-value assessed by paired *t*-test;

b Repeated measure ANOVA;

CI = confidence interval; MRS = Modified Ranking Scale;

* significant at *P* < 0.05
